# Minimal change disease associated with gastrointestinal stromal tumor accompanied by significantly elevated serum IgE level: a case report

**DOI:** 10.1186/s12882-022-02775-x

**Published:** 2022-04-11

**Authors:** Chun-Yang Yu, Jie Liu, Chang-Hai Qi, Zhen-Yu Wu, Yue-Fei Xiao, Xue-Guang Zhang

**Affiliations:** 1 Department of Nephrology, Aerospace Center Hospital, Beijing, 100049 China; 2 Health Management Center, Aerospace General Hospital, Beijing, China; 3 Department of Pathology, Aerospace Center Hospital, Beijing, China; 4 Department of Hepatological Surgery, Aerospace Center Hospital, Beijing, China; 5 Department of Nephrology, Capital Medical University Electric Teaching Hospital (State Grid Corporation of China Beijing Electric Power Hospital), Beijing, China

**Keywords:** Minimal change disease, gastrointestinal stromal tumor, Immunoglobulin E

## Abstract

**Background:**

Minimal change disease (MCD) is a common cause of the nephrotic syndrome. Several studies have shown an increased incidence of cancer in patients with MCD. However, there are no reports on the association between MCD and gastrointestinal stromal tumor (GIST).

**Case presentation:**

We report a case of a 66-year-old female with severe nephrotic syndrome and concomitant duodenal GIST. Immunoglobulin test showed a significant increase of IgE levels. The diagnosis of renal histopathology was MCD with subacute tubulointerstitial injury. The combination of preoperative Imatinib mesylate chemotherapy and tumor excision was accompanied by significant remission of proteinuria, and IgE level decreasing, without immunosuppressivetherapy.

**Conclusions:**

It is the first case report that MCD was associated with GIST and elevated IgE level. Clinically, in patients with elevated IgE level associated with nephrotic syndrome, the possibility of tumor must be taken into account when allergic factors are excluded.

## Background

Glomerular disease in malignant tumors has been recognized for decades. The most frequent types of malignant tumors are pulmonary, renal, and gastrointestinal solid tumors. Membranous nephropathy (MN) is the most common glomerulopathy associated with malignant tumors. However, other types have been reported, especially IgA nephropathy, minimal change disease (MCD), and anti-glomerular basement membrane (GBM) disease [1]. MCD is relatively uncommon.

In adults, MCD represents approximately 10-15% of patients with idiopathic nephrotic syndrome. Secondary MCD is involved in neoplasia, drugs (e.g., non-steroidal anti-inflammatory drugs), infections, and atopy. Frequently reported solid tumor associated with MCD are lymphoma, lung cancer, colorectal carcinoma, renal cell carcinoma, and thymoma. Pancreatic, breast, bladder, ovarian, and esophageal cancers have little correlation with MCD [2]. Here, we report a rare case of MCD associated with GIST and elevated serum IgE.

## Case presentation

**Fig. 1:**
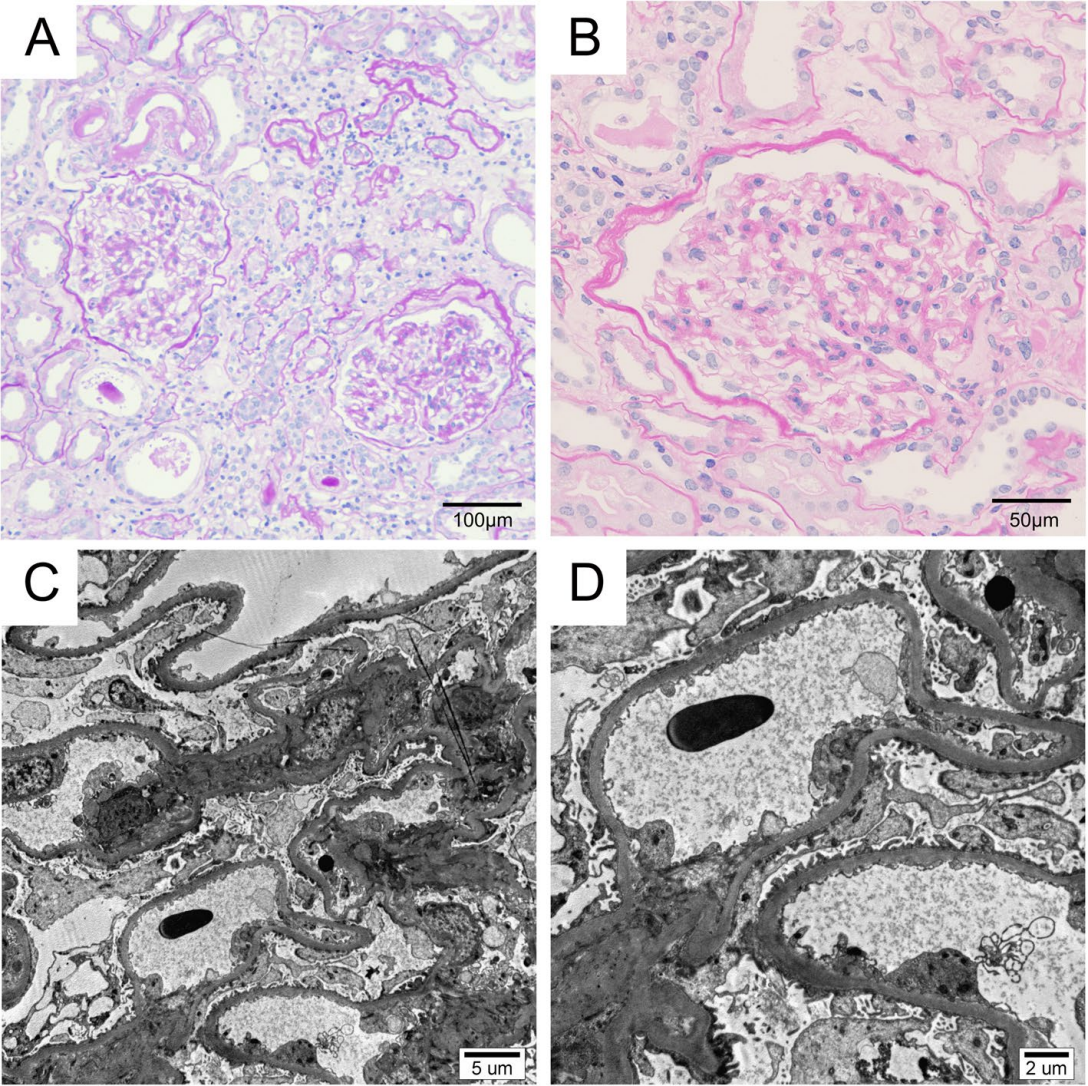
Kidney biopsy findings. **A** Appearance of glomeruli was essentially normal. Mild tubular atrophy, mild interstitial edema, inflammation and fibrosis in interstium, arteriole hyalinosis was observed (PAS stain, × 200). **B** No mesangial expansion and hypercelluarity was observed (PAS stain, × 400). **C **No mesangial matrix accumulation was observed and width of mesangium was normal (Electron microscopy, magnification × 3000). **D** No electron-dense deposit was found, the thickness of glomerular basement membrane (GBM) was normal (343 nm ~ 405 nm, average 382 nm), and footprocess of podocyte was widespreadly effaced (Electron microscopy, magnification × 6000)

A 66-year-old female was admitted to the hospital on October 26th, 2019, with edema of the eyelid and lower limbs for 1 month. 2 weeks ago, edema was aggravated, accompanied by increased foam in urine, nausea, abdominal distension, and poor appetite. Previous history: More than 10 years of type 2 diabetes, blood glucose was controlled satisfactorily. Hypertension has a history of more than 10 years, with poorly controlled blood pressure. A history of chronic bronchitis for many years. Physical examination disclosed an anemic appearance, moderate pitting edema of eyelids and lower limbs. Blood pressure was 176/92 mmHg, and serum glucose level was 5.4 mmol/L. Proteinuria was 22.224 g/24 h, serum albumin was 19.2 g/L, serum creatinine was 233.2 μmol/L, accompanied by hyperlipidemia (triglyceride: 10.10 mmol/L, Cholesterol: 13.65 mmol/L) and significantly increased erythrocyte sedimentation rate (ESR, 103 mm/h). Serum complement 3 (C3) level was in normal range, and C4 level was slightly high (41.2 mg/dL). Serum protein electrophoresis showed no M-spike. Coagulation function and thyroid function were normal. Antinuclear antibodies (ANA), anti-double-stranded DNA antibody, anti-neutrophil cytoplasmic antibody (ANCA) and anti-phospholipase A2 receptor (PLA2R) antibody were negative. Tests of hepatitis B surface antigen, antibodies against hepatitis C virus and human immunodeficiency virus were negative. Immunoglobulin examination revealed markedly elevated IgE level (7080 IU/mL, normal range 0-165 IU/mL), slightly decreased IgG level (505 mg/dL). IgA and IgM levels were normal. Mild abnormalities were found in serum light chain tests (κ chain: 114.69 mg/dL, λ chain: 64.41 mg/dL), but the ratio of κ to λ was normal (κ/λ:1.78). Both level of Ca125 and Ca199 were elevated (188.92 U/mL, and 48.65 U/mL, individually). Abdominal ultrasound indicated that the size and shape of the kidney was normal. There was a hypoechoic mass (5.0 cm × 3.0 cm × 4.1 cm) near the upper part of the right kidney. Computerized tomography (CT) scan for the abdomen revealed a space occupying lesion between the gastric antrum and the duodenum. Gastroscopy (November 13rd, 2019) showed that the proximal end of the posterior duodenal bulb had a raised lesion about 2.0 cm in size. The pathological result of gastroscope biopsy was inflammation. Kidney biopsy was performed. Of 10 glomeruli, 3 were globally ischemic sclerosed. Glomerular mesangium was normal. No nodular glomerulosclerosis, microaneurysm, hyaline insudation, crescent and capsule adhesion was seen. There was mild tubular atrophy, mild interstitial edema, inflammation and fibrosis in tubulointerstium. Tubular epithelial necrosis and brush border loss was occasionally observed. Arteries and arterioles exhibited mild intimal fibrosis and hyalinosis. Immunofluorescence microscopy for immunoglobulins G, A, and M, C3, C4, C1q, and fibrinogen was negative. It was observed by electron microscopy that there was no electron-dense deposits, the thickness of GBM was normal (343 nm ~ 405 nm, average 382 nm), and foot process of podocyte was widespreadly effaced. MCD and subacute tubulointerstitial injury was diagnosed (Fig. 1). The patient was discharged after remission under treatments including diuresis, antihypertensive treatment, blood glucose control, and anemia correction. We observed quickly decline of proteinuria (2.514 g/24 h), with serum albumin level elevated to 31.5 g/L, serum IgE level decreased to 6540 IU/mL and serum creatinine decreased to normal.

**Fig. 2:**
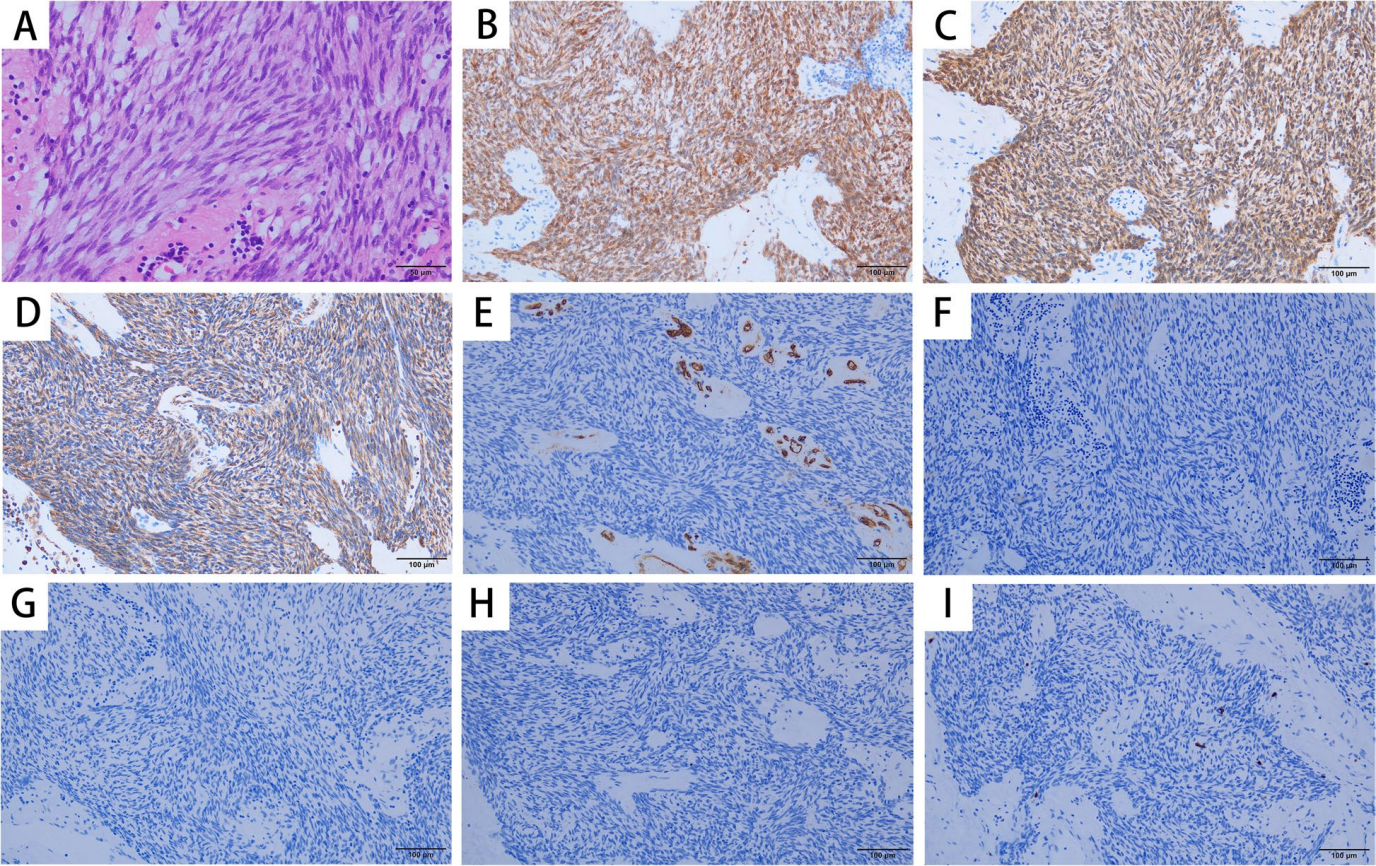
Tumor cells were spindle shaped cells with rod like hyperchromatic nuclei (**A**). Only one mitose was observed per 50 HPF (HE stain, × 400). The tumor cells were positive for CD117 (× 400) (**B**), Dog-1 (**C**) and Vimentin (**D**), while stains for CD34 (**E**), smooth muscle actin (SMA) (**F**), desmin (**G**) and s-100 (**H**) were negative. The positive ratio of Ki-67 staining was less than 10% (**I**)

**Fig. 3:**
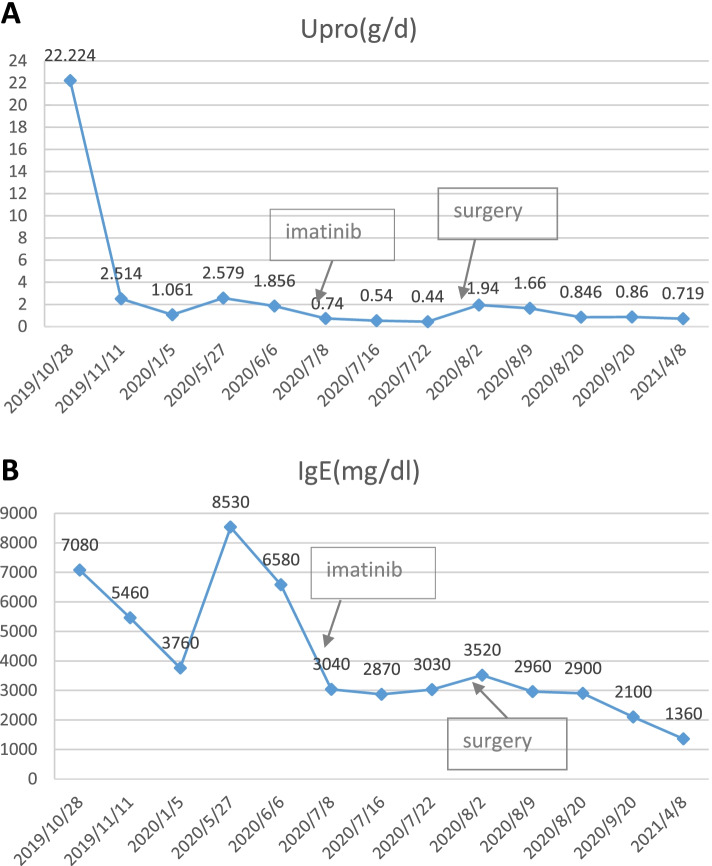
Proteinuria (**A**) and serum IgE level (**B**) declined with imatinib treatment and surgical removal of the tumor

On May 10th, 2020, the patient was readmitted for review. Proteinuria was 2.579 g/24 h and serum albumin was 34.1 g/L. But the serum IgE level went up to 8530 IU/mL. Abdominal enhanced magnetic resonance imaging (June 2nd, 2020) disclosed that the intestinal wall of the descending junction area of the duodenum was thickened, with a range of about 47 mm × 61 mm, lymphoma was not excluded. Electronic ultrasound endoscopy was performed again on June 8th, 2020. A 1.0 cm × 1.5 cm deep ulcer was found in the upper part of the descending duodenum. The tumor was located in the posterior lateral wall of the descending duodenum, with a range of about 4.5 cm × 3 cm × 3 cm. The pathologic diagnosis of the tumor was duodenalgastrointestinal stromal tumor (GIST), spindle cell type, low-risk type. Only one mitose was observed per 50 HPF (Fig. 2A). Immunoperoxidase staining for CD117 (c-kit), Dog-1, and Vimentin were positive in the tumor cells (Fig. 2B), while staining for CD34, smooth muscle actin (SMA), desmin and S-100 were negative. The positive ratio of Ki-67 staining was lower than 10%. On June 24st, 2020, the patient was administrated with imatinib mesylate (400 mg/day), and the surgical excision of the tumor was performed after 1 month. Imatinib mesylate has been administered subsequently. The histopathological result of the tumor was also duodenal GIST (spindle cell type, and low-risk type). The patient had never received any specific immunosuppressive therapy for proteinuria. Proteinuria slowly declined to 0.846 g/d 1 month after surgery, with serum albumin increased to 36.4 g/L and IgE decreased to 2900 IU/mL. 2 months after surgery, proteinuria was 0.86 g/d, serum albumin was 36.3 g/L and serum IgE was 2100 IU/mL. Eight months after surgery, proteinuria was 0.719 g/d, serum albumin was 45.3 g/L (Fig. 3A) and serum IgE was 1360 IU/mL (Fig. 3B).

## Discussion and conclusion

The clinical manifestation of this patient was nephrotic syndrome with a history of diabetes, and the possibility of diabetic nephropathy was considered at admission. However, the kidney biopsy confirmed MCD, which can be caused by a variety of factors, such as allergy, tumor, drugs, and etc [2].

Imaging examination of the patient indicated that a space occupying lesion was in the duodenum. GIST was confirmed by pathological examination after surgical resection. Previously, two cases of nephrotic syndrome caused by GIST have been reported, one of which was confirmed to be MN by renal biopsy [3] and the other was not subjected to renal biopsy [4]. MCD in this case may be caused by GIST, and it is speculated that some tumor factors secreted by GIST may cause the increased permeability of the glomerular basement membrane (GBM), then lead to proteinuria [5, 6].

Another finding in this case was a significant increase in serum IgE level, and a significant decrease in serum IgE level and remission of the nephrotic syndrome after surgical resection of the tumor. It was suggested there is a correlation among elevated IgE level, GIST and MCD. With the deepening of IgE related studies, not only IgE is the relevant indicator of anaphylaxis, but also its correlation with tumors has increasingly become a research hotspot [7]. In the human body, tumor pathogenic factors and tumor cells act as a foreign antigen or allergen, constantly stimulate the body to generate antibodies, including IgE antibodies and forming a symptom similar to allergic reaction [8]. In recent decades, evidences have emerged relating allergies with cancer development [9]. However, most of the results of epidemiology studies have been controversial. One indicate that allergies can reduce the risk of cancer [10, 11], another indicate that they may increase this risk [12, 13]. In any cases, IgE production involves activation of helper T cell subsets 2 (Th2) to generate interleukin-4 (IL-4) and interleukin-13 (IL-13), which stimulate B lymphocytes to synthesize IgE [14, 15]. IgE acts on mast cells and basophil cells to release histamine and leuktriene [16, 17], and acts on monocyte and macrophage to release tumor necrosis factor α (TNF-α) [18], which might enhance permeability of the GBM. In addition, IL-13 can also cause the disorder of podocyte skeleton protein and affect the function of foot process, which might be involved in proteinuria [19, 20].

In conclusion, for patients with elevated IgE level associated with nephrotic syndrome, the possibility of tumor must be taken into account when allergic factors are excluded.

## Data Availability

Not applicable.

## Abbreviations

MCD: Minimal change disease
GIST: Gastrointestinal stromal tumor
MN: Membranous nephropathy
ANA: Antinuclear antibodies
ANCA: Antineutrophil cytoplasmic antibody
PLA2R: Phospholipase A2 receptor
CT: Computerized tomography
SMA: Smooth muscle actin
GBM: Glomerular basement membrane
TNF-α: Tumor necrosis factor α
